# Web-Based Guidance Through Assisted Reproductive Technology (myFertiCare): Patient-Centered App Development and Qualitative Evaluation

**DOI:** 10.2196/25389

**Published:** 2021-08-03

**Authors:** Ellen Marie Sparidaens, Rosella P M Hermens, Didi D M Braat, Willianne L D M Nelen, Kathrin Fleischer

**Affiliations:** 1 Department of Obstetrics and Gynaecology Radboud University Medical Center Nijmegen Netherlands; 2 Scientific Institute for Quality of Healthcare (IQ Healthcare) Radboud University Medical Center Nijmegen Netherlands; 3 The Fertility Partnership Center of Reproductive Medicine Düsseldorf Germany

**Keywords:** eHealth, infertility, interactive, mobile apps, patient education, patient-centered care, personalized, topic

## Abstract

**Background:**

Providing patient-centered fertility care is known to improve quality of life and can reduce anxiety and depression. In a previous study, we established the need for a web-based app providing personalized information and interactive functionalities among couples undergoing intracytoplasmic sperm injection with surgically retrieved sperm.

**Objective:**

This study aimed to design, develop, and qualitatively evaluate a multifaceted web-based app for infertile couples undergoing intracytoplasmic sperm injection with surgically retrieved sperm during their treatment trajectory.

**Methods:**

The web-based app was developed in three phases: (1) we established a patient-centered functional design, (2) developed the app in collaboration with medical and technical professionals, and (3) qualitatively evaluated the app among couples using a think-aloud method.

**Results:**

The basis of the app is the couple’s visualized treatment trajectory. The app provides personalized and interactive functionalities; for example, customized information and communication options. During qualitative evaluation, myFertiCare was highly appreciated and received a median score of 8 out of 10. The main improvements made upon conclusion of the think-aloud sessions were related to faster login and easier app navigation.

**Conclusions:**

A patient-centered web-based app aimed at guiding couples through their fertility treatment course was systematically designed, developed, and positively evaluated by patients and medical and technical professionals.

## Introduction

According to the Institute of Medicine, health care should be safe, effective, timely, efficient, equitable, and patient-centered [1]. Patient-centeredness is described as “providing care respectful of and responsive to individual patient preferences, needs and values, and ensuring that patient values guide all clinical decisions” [1]. Providing patient-centered fertility care can improve quality of life and can reduce anxiety and depression [2]. It has been reported that patient-centeredness in fertility care needs improvement [3,4]. Crucial aspects of patient-centeredness in fertility care are provision of adequate information, continuity of care, and active involvement of patients in their treatment course [5-8]. The internet is known to be an important source of information and support for infertile couples [9-11]. A possible instrument for improving patient-centeredness is the use of eHealth tools; that is, web-based apps in health care [12]. Previous eHealth initiatives in fertility care aimed mainly at information provision, support, and mental health promotion [13]. These initiatives only contained few interactive web-based components. Aarts et al [13] concluded that these initiatives could be improved by including more interactive and dynamic elements as their key components. Infertile couples are known to specifically prefer personalized information and appreciate being able to communicate with both their treatment team and fellow patients [3,10]. eHealth tools are a promising strategy to empower patients in managing their own treatment trajectory.

In a previous study [14], we established the need for a web-based app providing personalized information and interactive functionalities among couples undergoing intracytoplasmic sperm injection (ICSI) with surgically retrieved sperm. We hypothesized that a web-based app is specifically suitable for this group of patients because of the psychological and physical burden of the multidisciplinary treatment on both partners. Therefore, the aim of this study was to design, develop, and qualitatively evaluate a multifaceted web-based app for use by couples undergoing ICSI with surgically retrieved sperm during their treatment trajectory.

## Methods

### Systematic Approach

myFertiCare was developed in three phases: (1) we established a patient-centered, functional design for the app; (2) developed myFertiCare in collaboration with medical and technical professionals; and (3) had myFertiCare qualitatively evaluated for usability, with a think-aloud method.

### Phase 1: Establishment of a Patient-Centered Functional Design

The functional design of myFertiCare is based on (1) literature review; (2) interviews with an expert panel comprising a gynecologist, a urologist, an embryologist, an expert in patient-centered innovation, and a board member of Freya, the Dutch association for infertility problems; and (3) interviews with a patient panel. This was part of our previous study [14] on the informational needs of couples undergoing ICSI with surgical sperm retrieval. The patient panel consisted of 11 couples, a number that was determined through data saturation. We conducted semistructured interviews with each couple individually. The data were analyzed using a constant comparative method. The functional design that followed from this process was verified by the clinic’s fertility treatment team and supplemented with their suggestions. Both the expert and the patient panels were enthusiastic about the idea of a web-based app to guide couples through their treatment trajectory. The overall opinion was that the more functionalities an app provides, the better the app, so that people are motivated to use it. The participants specifically valued personalized and interactive functionalities. Various functionalities were suggested, such as being able to view appointments, test results, and information about lifestyle advice; information about the clinic’s fertility treatment team; and communication with physicians and peers. The interviewees also highlighted the safeguarding of confidential information, which they stated should be at the core of app development [14].

### Phase 2: Development of myFertiCare

Based on the preferences of the expert and the patient panels, myFertiCare was developed in close collaboration with medical professionals from the department of Reproductive Medicine, Radboud University Medical Center (RUMC), and technical experts from a Dutch company specializing in eHealth. Together, they formed the project team. Development was an iterative process. The desired functionalities of myFertiCare were categorized by the medical professionals as must-have functionalities that had to be available before the app could be made available on the internet, or as nice-to-have functionalities that could be developed later. Subsequently, the must-have functionalities were developed by the technical experts and tested in a test environment by both the technical experts and the medical professionals. Technical adjustments were made as necessary, and the testing cycle was started over again. Once all the must-have functionalities were developed and tested by the technical and medical experts, myFertiCare was made available through the hospital website, the App Store, and Google Play Store to couples undergoing ICSI with surgical sperm retrieval. myFertiCare was also incorporated in the existing hospital information systems. After the app was live, all the nice-to-have functionalities were developed through the same development cycle. After each functionality was iteratively developed and tested, it was immediately made available to all myFertiCare users. The duration of the whole development trajectory was approximately 1.5 years.

### Phase 3: Qualitative Evaluation of myFertiCare for Usability Through the Think-Aloud Method

After all the must-have and nice-to-have functionalities were implemented, we began the qualitative evaluation of myFertiCare. In total, 21 couples, who consecutively visited the fertility clinic, were invited by their physicians to participate in the think-aloud sessions. Six couples agreed to participate, which accounted for 9 participants (4 men and 5 women) (Tables 1 and 2). Reasons for nonparticipation were being too busy, already having too much going on, or simply not wanting to participate. Think-aloud is a research method in which participants verbalize their thought processes while interacting with a tool [15]. It provides a valid source of data about participants’ thought processes and can be used effectively in qualitative studies [15]. Our aim was to identify usability flaws and to provide suggestions for design modification.

**Figure 1:**
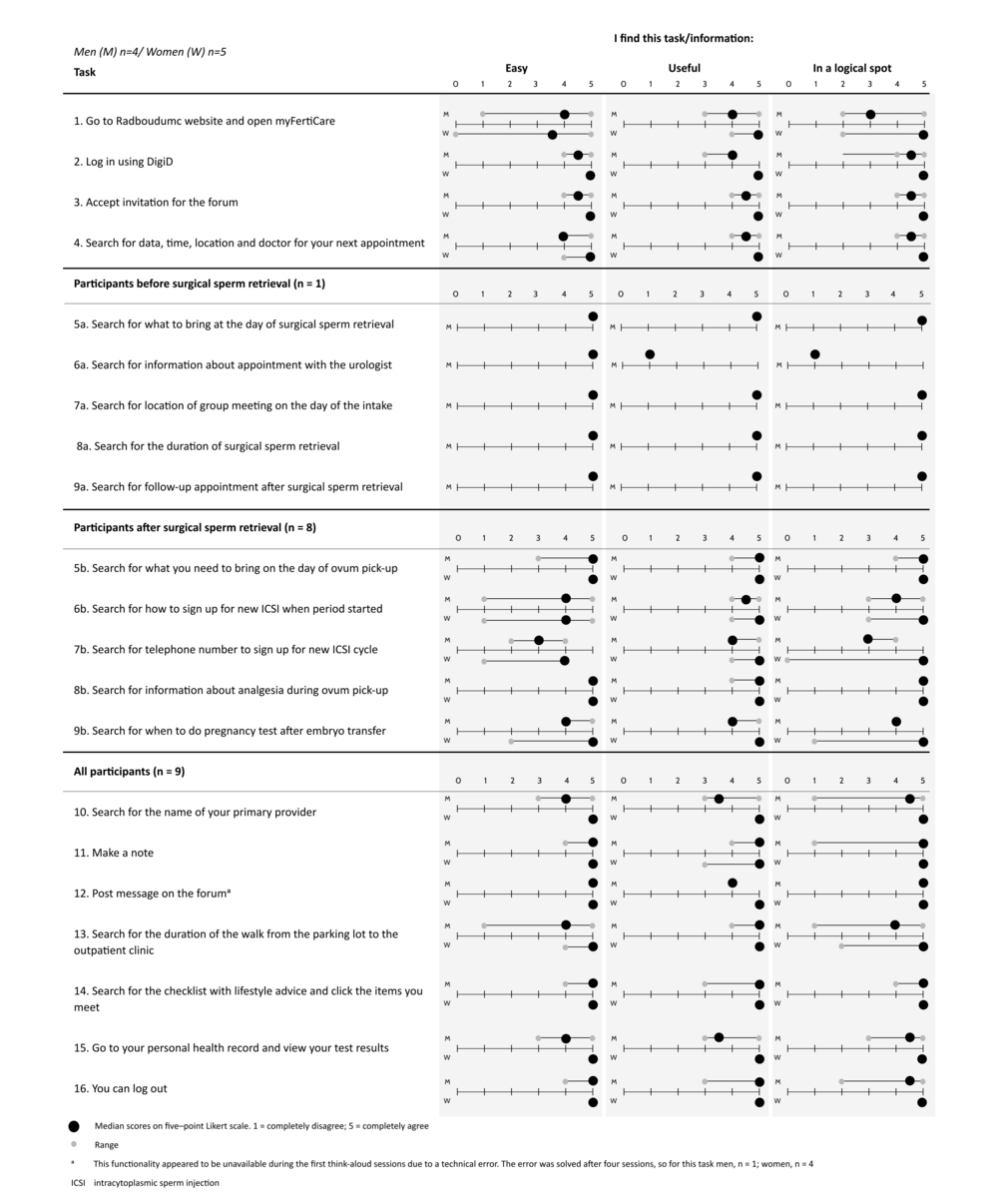
Results obtained from think-aloud sessions.

The participants were individually provided with 16 tasks to perform using myFertiCare while thinking out loud (Figure 1). Of these tasks, 11 were the same for every participant and 5 focused on the specific phase of treatment that an individual was in. By completing these tasks, the participants explored all the functionalities of myFertiCare. The researcher observed the participants and asked questions for clarification where needed. The researcher took notes, also focusing on nonverbal communication of participants. In addition, the sessions were audio-taped. After completing each task, the participants answered 3 task-linked questions (“I find this task easy,” “I find this information useful,” and “I find this information is in a logical spot”) with a 5-point Likert scale to rate responses. They could also add free comments.

**Figure 2:**
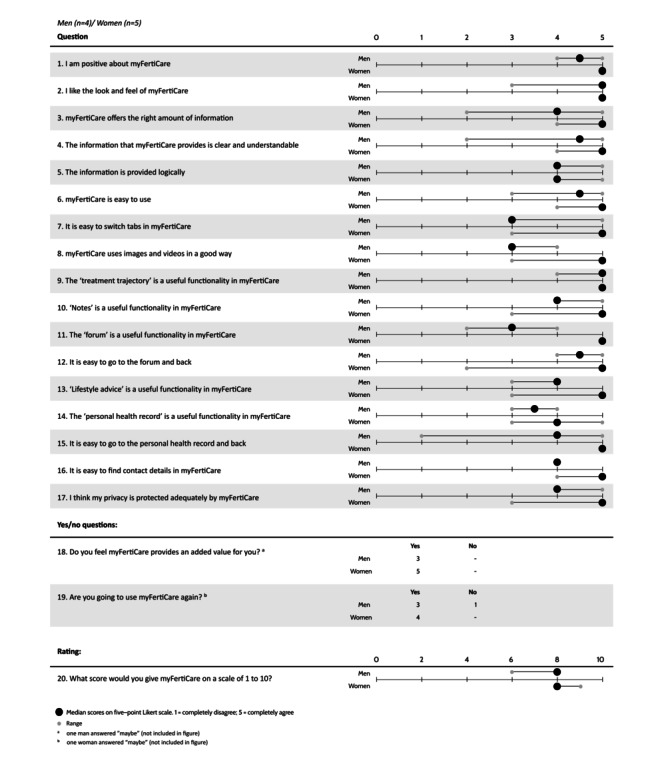
Results obtained from the questionnaire on users' experience with myFertiCare.

Immediately after each think-aloud session, the participants completed a self-developed questionnaire on their experiences using myFertiCare (Figure 2). The questions were about participants’ attitudes toward usability, privacy, understandability of information, and the usefulness of various functionalities of myFertiCare. The questionnaire consisted of 20 questions: 17 with responses rated on a 5-point Likert scale, 2 to be answered with “yes” or “no,” and 1 open question. Again, the participants could also write free comments.

**Table 1:** Demographic characteristics of the study participants (N=9).

Characteristic		Men (n=4)	Women (n=5)
Age (years), median (range)		33 (27-47)	30 (28-36)
Daily internet usage in a private setting (minutes), median (range)		60 (45-60)	90 (60-180)
Treatment-related use of the internet, n		3	5
Use of myFertiCare prior to the think-aloud session, n		1	2
**Educational status,^a^ n**			
	Low	0	0
	Medium	1	2
	High	3	3
**Ethnic background, n**			
	Caucasian	3	4
	Non-Caucasian^b^	1	1
**Those who already have children, n**			
	Yes	0	0
	No	4	5

^a^Low educational status includes no education and lower general secondary education. Medium educational status includes higher general secondary education and intermediate vocational education. High educational status includes higher vocational education and a university degree.

^b^One man from Indonesia and 1 woman from Suriname.

**Table 2:** Demographic characteristics of the couples who participated in the study (N=6).

Characteristic		Couples
**Socioeconomic status,^a^ n**		
	Low	3
	Medium	3
	High	0
**Stage of treatment, n**		
	Before surgical sperm retrieval	1
	After surgical sperm retrieval and before ICSI^b^	2
	During first ICSI cycle	2
	After at least one full ICSI cycle	1
Duration of infertility (months), median (range)		28 (16-47)

^a^According to the Dutch Social and Cultural Planning Office: Low socioeconomic status included a status score of ≤–1; medium socioeconomic status included a status score between –1 and +1; high socioeconomic status included a status score of >1.

^b^ICSI: intracytoplasmic sperm injection.

The think-aloud sessions were conducted until saturation was reached. The duration of a session was approximately 15 to 20 minutes. The audio-taped sessions were transcribed verbatim. Data were analyzed anonymously. An open coding method was applied. We coded quotes that identified usability flaws or provided suggestions for modification of the app’s design. A second researcher verified the coding process. Differences were discussed until consensus was reached. Ethical approval was proposed but was not required according to the local research ethics committee (CMO Arnhem Nijmegen, file# 2016-2485). All participants provided written informed consent.

## Results

### Phases 1 and 2: Design and Development of myFertiCare

**Figure 3:**
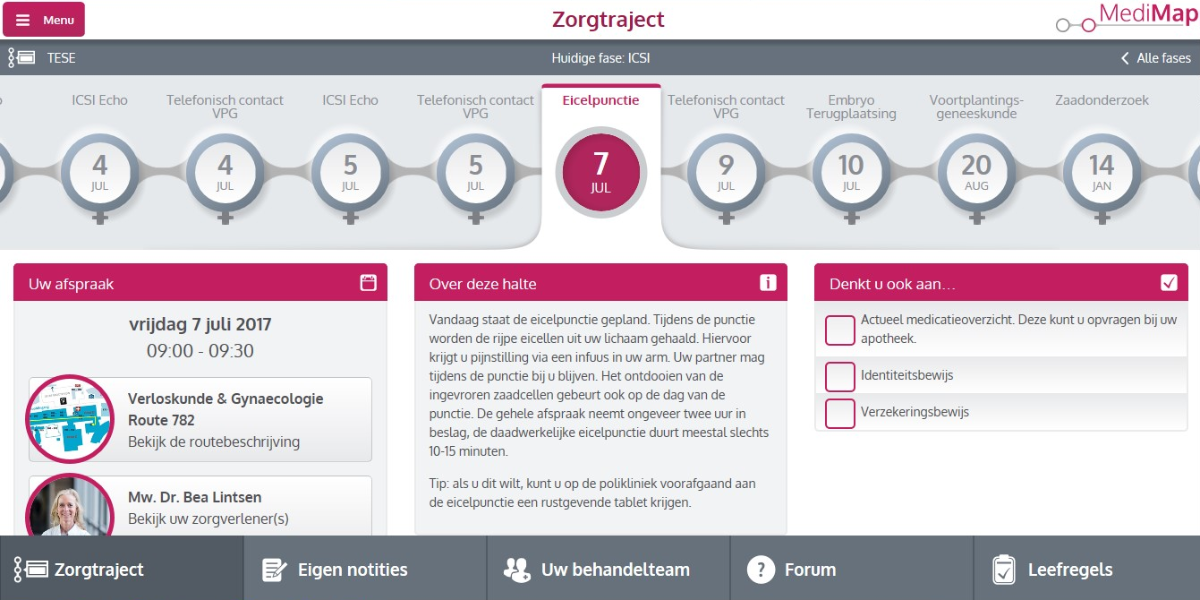
Screenshot of the myFertiCare app.

Based on the results of phase 1 [14], myFertiCare was developed as a web-based app available on the RUMC website, Google Play Store, and Apple App Store for couples undergoing ICSI with surgical sperm retrieval at the RUMC. Patients log in using their digital identity (DigiD), which is provided by the government of the Netherlands to assure safe access to all governmental institutions. This guarantees the safety of couples’ medical data. The apps of both partners are synchronized, so that individuals can also see their spouse’s information. myFertiCare is free for use and is offered in addition to usual care. A screenshot of the app is provided in Figure 3.

myFertiCare contains personalized and interactive functionalities that are divided over 5 tabs:

1. Treatment trajectory: this is the basis of the app. The treatment trajectory is visualized as a subway map in which every stop stands for one of the appointments a couple must have in order to move forward. Couples can see their past appointments and future scheduled appointments with the corresponding data, time, physician, and location, but they can also see future appointments that are not scheduled yet. Thus, couples are better prepared for the upcoming trajectory and know what to expect. Each stop on the subway map provides information about the specific phase of the treatment trajectory and provides advice on how to prepare for the appointment, and, if applicable, informs them of anything they should bring with them for the appointment. Furthermore, users receive support messages before or after certain appointments to comfort them or provide some advice. These support messages are sent via the app or via text message.
2. Notes: users can write notes that are synchronized with their spouse’s notes. For example, couples can compose a topic list with questions they want to ask during their upcoming physician’s appointment.
3. Care providers: an overview of the whole treatment team is provided through photographs, with an individual’s primary care provider on top. Users can ask medical questions to the treatment team, and they are answered within 24 hours.
4. Forum: patients can communicate with peers on the forum. The forum is supervised by a clinician.
5. Lifestyle advice: this is provided as separate checklists for men and women. The aim is to improve treatment outcomes; that is, to improve the chance of retrieving semen through percutaneous epididymal sperm extraction or testicular sperm extraction and concomitantly the probability of conception. Users can click the boxes of the checklist, which are also synchronized with those of their partner.

In addition to the 5 tabs, myFertiCare provides a main menu with general information (eg, contact details and app settings) and a link to the user’s personal health record. In the personal health record, users can see their own test results and read the correspondence between their primary care provider and their family physician.

Finally, for couples who are not yet being treated at RUMC and thus do not have login details, a preview version of myFertiCare is available. In this version, they can view the general treatment trajectory and consult the checklist with lifestyle advice. Thus, they can prepare themselves for their intake appointment.

### Phase 3: Qualitative Evaluation of myFertiCare for Usability With the Think-Aloud Method

The think-aloud sessions yielded both positive and negative feedback. Given the aim of the study, we focused on opportunities to improve the app. As described earlier, every participant completed 16 tasks (11 generic and 5 personalized). This resulted in 21 different tasks. After each task, the participants answered 3 task-linked questions (“I find this task easy,” “I find this information useful,” “I find this information is in a logical spot”) on a 5-point Likert scale (1=“totally disagree,” 5=“totally agree”). Figure 1 shows a summary of the results. In general, participants considered the tasks (ie, the functionalities that myFertiCare provides) easy and useful. They also considered that the information was provided logically.

Although the scores for all tasks were high, the participants named some discomforts and suggestions for improving the app design. They commented that logging in with their DigiD was too cumbersome, since it consists of a username, password, and verification via text message. It was also noted that moving along the visualized treatment trajectory was difficult. The participants attempted to slide through the treatment trajectory, which was not possible. Instead, they had to click on stops to move to this specific stop. Furthermore, they noticed that the app did not open with the most recent appointment, which was the mode they preferred. When using the forum, participants regretted that they could not delete an erroneous message they had posted. Finally, participants expressed the need for a home button to lead them to the home screen of the app.

At the end of each think-aloud session, the participants completed a questionnaire about their experience using myFertiCare. Figure 2 shows a summary of the results. The participants allocated high scores to all surveyed items that related to usability, understandability of information, the usefulness of various functionalities, and privacy. The men were consistently slightly more critical than the women. The space for writing free comments revealed no additional information. All participants felt that myFertiCare provides an added value to them. All but 1 participant intended to use myFertiCare in the future. In conclusion, myFertiCare was rated 8 out of 10 (Figure 2).

Guided by the think-aloud sessions, we made various improvements in app design. We made it possible for myFertiCare users to create a 4-digit entry code after the first login with DigiD, so that fast but equally safe access was enabled for future use. Furthermore, opening myFertiCare with the most recent appointment was made possible, while proceeding through the treatment trajectory. We added an option to remove a message from the forum after it has been posted as well. A home button was incorporated, which leads users to the app’s home screen.

## Discussion

### Principal Findings

We designed, developed, and qualitatively evaluated an eHealth app for fertility care in accordance with a methodological framework, based on couples’ information needs and input provided by health care providers. The basis of the app is the visualized treatment trajectory. The app provides both personalized and interactive functionalities, including customized information and communication options. On thorough qualitative evaluation, myFertiCare received high usability ratings. The participants felt that myFertiCare provides an added value during their treatment. The app was rated with a median score of 8 out of 10. The most important improvements after the think-aloud sessions were related to faster login and easier navigation through the app.

A large part of research in fertility care is aimed at the female partner. We chose to include both partners when developing the app, since it is recognized that men should have a well-defined role as an equal partner during fertility treatment, particularly in cases of male infertility [16]. A previous study by Sylvest et al [11] reported that men registered a marked time lag between diagnosis and treatment initiation. They felt “they were in a maze without a map” and expressed the need for detailed information about the treatment plan, including a timetable, so that they could control and manage their lives [11]. With a visualized treatment trajectory as the basis of myFertiCare, we aimed to meet this need and guide couples through their entire treatment trajectory. In our opinion, patient satisfaction with information provided by the clinic is an important indicator of the quality of fertility care, although in fertility care, the focus is often on live birth rates. Alper et al [17] further endorsed this idea.

There is literature available on eHealth initiatives in fertility care [13], primarily on online support groups. In general, there is a lack of initiatives that provide interactive and dynamic elements, and there is a lack of methodological standards for these complex interventions [13]. There has been 1 web-based initiative that provides both information and peer support, which showed high patient appreciation [18]. Furthermore, a web-based community has been reported in which couples could communicate and share information with professionals and peers [19].

Compared to previous initiatives, a strength of myFertiCare is that it provides a large variety of personalized and interactive functionalities centered around the visualized treatment trajectory of the couple. Another methodological strength of our study is its 3-phase systematic approach: first, a functional design for the app was developed through a qualitative assessment of the informational needs of patients; second, myFertiCare was actually developed; and third, myFertiCare was qualitatively evaluated for usability through the think-aloud method. All 3 phases were carried out in collaboration with patients and medical and technical professionals, which is important for successful eHealth development and implementation [20]. Our qualitative evaluation of myFertiCare for usability is crucial since usability evaluations are critical to the success of adopting an interactive eHealth app [20,21]. The think-aloud method is preferred for uncovering usability problems, and it provides understanding of how users interact with myFertiCare [22]. Furthermore, the think-aloud method is especially suitable since we used both a concurrent method (ie, while performing the task) and a retrospective method (ie, immediately after performing the task) to report participants’ thinking, a method that has been suggested for producing optimal data quality [23].

### Limitations

Our study also has some limitations. It could be argued that the study population for the think-aloud sessions was relatively small. However, studies have shown that for a think-aloud test, 5 participants are enough for success in identifying usability problems in a user interface [24]. Since we included 9 participants and achieved data saturation, we are confident that we have identified all the possible usability problems. Furthermore, it is known to be a challenge for a researcher to remain consistent when it is necessary to intervene in a think-aloud session; for example, when a participant is unable to complete a task, clarification on a participant’s comment is required, or a participant sidesteps the functionality of interest [25]. In these situations, it is important to explain to a participant that it is the aim of the study to identify problems and to invite them to approach the problem otherwise. It has been reported that researchers often unintentionally intervene in theoretically inconsistent ways [25]. We made a conscious effort to achieve a reliable data set by being aware of these limitations and through triangulation of research methods (namely the think-aloud, task-linked questions, and researcher’s observations) and the recording, transcribing, and coding of the interviews.

### Practical Implications

This study provides a framework for patient-centered design, development, and evaluation of an eHealth app. Our systematic approach, in which patients and professionals participated in every phase of the process, is particularly suitable in the current era where patient-centeredness is highly valued. Furthermore, we obtained insight into the various functionalities that patients appreciate in a web-based app. The framework we developed for myFertiCare supports professionals in fertility care for guiding patients through their treatment trajectory and delivering patient-centered care. In the near future, myFertiCare will also be evaluated quantitatively. Expansion of eHealth tools to cover the whole fertility care journey and expansion to other medical disciplines is considered of high value. Development of eHealth tools from a patient’s viewpoint is an opportunity to empower patients in managing their own treatment trajectories in the current era of patient-centered care.

### Conclusions

We designed, developed, and qualitatively evaluated a multifaceted web-based app, myFertiCare, through a systematic approach in which patients and medical and technical professionals participated in every phase. This app aims to guide couples undergoing ICSI with surgically retrieved sperm through their treatment trajectory. myFertiCare provides personalized and interactive functionalities, facilitating the provision of patient-centered care and empowering patients to manage their own treatment trajectory. The app had a high usability rating and was highly appreciated by both male and female partners.

## Abbreviations

DigiD: digital identity
ICSI: intracytoplasmic sperm injection
RUMC: Radboud University Medical Center
